# *Redondoviridae* and periodontitis: a case–control study and identification of five novel redondoviruses from periodontal tissues

**DOI:** 10.1093/ve/veab033

**Published:** 2021-04-12

**Authors:** Yu Zhang, Chunmei Wang, Xiping Feng, Xi Chen, Wen Zhang

**Affiliations:** Department of Preventive Dentistry, Shanghai Ninth People's Hospital, College of Stomatology, Shanghai Jiao Tong University School of Medicine, Shanghai, China; National Clinical Research Center for Oral Diseases, Shanghai, China; Shanghai Key Laboratory of Stomatology & Shanghai Research Institute of Stomatology, Shanghai, China; Shanghai Veterinary Research Institute, Chinese Academy of Agricultural Sciences, Shanghai, China; Department of Preventive Dentistry, Shanghai Ninth People's Hospital, College of Stomatology, Shanghai Jiao Tong University School of Medicine, Shanghai, China; National Clinical Research Center for Oral Diseases, Shanghai, China; Shanghai Key Laboratory of Stomatology & Shanghai Research Institute of Stomatology, Shanghai, China; Department of Preventive Dentistry, Shanghai Ninth People's Hospital, College of Stomatology, Shanghai Jiao Tong University School of Medicine, Shanghai, China; National Clinical Research Center for Oral Diseases, Shanghai, China; Shanghai Key Laboratory of Stomatology & Shanghai Research Institute of Stomatology, Shanghai, China; School of Medicine, Jiangsu University, Zhenjiang, China

**Keywords:** periodontitis, Redondoviridae, epidemiological study, novel redondoviruses

## Abstract

*Redondoviridae* is a family of DNA viruses recently identified in the human oro-respiratory tract. However, the characteristics of this new virus family are not yet fully understood. The aim of the present study was to investigate the relationship between redondoviruses and chronic periodontitis. In addition, the complete circular genome, phylogenetic relationship, and biological characteristics of novel redondoviruses were analyzed. The gingival tissues of healthy individuals (*n* = 120) and periodontitis patients (*n* = 120) were analyzed using nested polymerase chain reaction assays. The prevalence of redondovirus infection in the periodontitis group was 71.67%. Logistic regression analysis revealed an association between redondoviruses and chronic periodontitis after controlling the confounding factors (odds ratio = 2.53). Five novel redondoviruses, named ‘human periodontal circular-like virus (HPeCV)’, were identified in patients with periodontitis and detailed genetic analysis of the viruses was performed. The 3,035–3,056 bp genome contained a capsid protein, a replication-associated protein, an open reading frame 3 protein, and a stem-loop structure. Phylogenetic analysis demonstrated that HPeCV-1, HPeCV-10, and HPeCV-25 formed a cluster. Recombination may be common in the genomes of HPeCVs. Potential antigenic epitopes in the capsid protein, which may be involved in the host immune response, were predicted. In conclusion, periodontitis patients had a significantly higher prevalence of redondoviruses than healthy controls. Genetic characterization enhanced the current understanding of the genetic diversity and pathogenicity of redondoviruses as well as their association with periodontitis in humans. The data presented in this article will expand the current understanding of the epidemiology, genetic diversity, and pathogenicity of redondoviruses.

## 1. Introduction

Periodontitis is a highly prevalent oral disease (Qasim et al. 2020) characterized by inflammation of the periodontal tissues, progressive destruction of the tooth attachment apparatus, and resorption of the alveolar bone (Klokkevold 2006). Severe periodontitis may lead to tooth mobility or loss (Petersen and Baehni 2012). Approximately four billion individuals worldwide have a history of periodontitis and the global prevalence of severe periodontitis is estimated to be 11 per cent (Marcenes et al. 2013). The recent fourth National Oral Health Survey in China revealed that periodontal diseases were common among Chinese adults aged from 35 to 44 years (Sun et al. 2018). In addition, severe periodontitis was listed as the sixth most prevalent pathology, affecting more than 740 million individuals (Kassebaum et al. 2014; Jin et al. 2016; Tonetti et al. 2017).

The etiology of periodontitis is multifactorial and involves complex interactions between the oral microbiome and host immune system. Microbial interactions are implicated in the onset of periodontitis, especially the development of severe periodontitis (Meyle and Chapple 2015; Slots and Slots 2019). Viruses have shown emergent pathogenic roles, either through the host immune cells or as co-infectors with bacteria to deregulate the host-defense systems (Meyle and Chapple 2015; Slots and Slots 2019).

We previously analyzed the human gingival tissue virome in healthy individuals and periodontitis patients based on viral metagenomics. We identified several novel anelloviruses and bacteriophages associated with periodontitis (Zhang et al. 2016; Zhang et al. 2017; Zhang et al. 2019), alerting us that there were more undiscovered human viruses associated with periodontitis that required further investigations. Abbas et al. (2019) recently identified a new family of human DNA viruses named ‘*Redondoviridae*’ using metagenomics. Nineteen redondovirus genomes were recovered from thousands of metagenomic samples simultaneously. Redondoviruses are tiny, circular, single-stranded DNA viruses that encode a capsid protein (Cap) and a replication initiation protein (Rep). They are considered a new group of circular Rep-encoding single-stranded DNA (CRESS) viruses (Rosario et al. 2012) due to the low identity to other identified CRESS viruses. Based on the protein diversity of viral Rep, *Redondoviridae* is grouped into two genera: Vientoviruses and Brisaviruses. *Redondoviridae* is reported to be the second most prevalent eukaryotic DNA virus family in humans, mainly colonizing the oropharynx and respiratory tract (Abbas et al. 2019). Notably, the analysis of three previous study metadata suggested the possibility of an association between redondovirus sequences and periodontal diseases (Wang et al. 2013; Shi et al. 2015; Califf et al. 2017). However, the sample sizes were limited in these studies (16, 12, and 2, respectively); therefore, further research exploring the association with periodontitis is required. To date, there are no confirmed genotypes and the genetics and pathogenesis of the new virus family are not fully understood. Therefore, the aim of the present study was to investigate the relationship between redondoviruses and periodontitis. In the epidemiological investigation, we also identified five novel viruses from the gingiva of chronic periodontitis patients that belong to the new *Redondoviridae* family. In addition, the complete circular genome, phylogenetic relationship, and biological characteristics of the redondoviruses were investigated.

## 2. Materials and methods

### 2.1 Clinical sample collection

The present study was conducted at the Ninth People’s Hospital, College of Stomatology, Shanghai Jiao Tong University School of Medicine, Shanghai, China. The study was approved by the institutional ethical review board at the Ninth People’s Hospital, Shanghai Jiao Tong University School of Medicine, China (Ref No: 2018217) and procedures were carried out according to the guidelines of the Declaration of Helsinki. All participants signed an informed consent form. Adult participants aged 18–65 years (*n* = 240) were recruited including volunteers with healthy periodontium (*n* = 120) and chronic periodontitis patients (*n* = 120). Individuals suffering from systemic diseases (such as kidney or liver disorders, cardiovascular disease, diabetes mellitus, human immunodeficiency virus infection) or malignancies and those who were pregnant were excluded. In addition, individuals who reported a history of periodontal treatment, smoking (>15 cigarettes/day), or antibiotics treatment (during the previous 6 months) were excluded. Clinical examination was performed for all participants and the periodontal health was evaluated including the pocket depth (PD), bleeding on probing (BOP), plaque index (PLI), gingival index (GI), and clinical attachment loss (CAL). The extent of alveolar bone resorption was determined radiographically using an orthopantomogram.

Participants who had BOP (+), mean PD ≥ 6 mm, mean CAL ≥ 3 mm, and alveolar bone resorption were placed in the chronic periodontitis group. Participants without any signs of gingivitis, BOP, and CAL, with mean PD ≤ 4 mm were placed in the periodontally healthy group. Biopsy samples from patients in the chronic periodontitis group were collected from the sulcular region including the gingival epithelium and connective tissues facing towards the sulcus while performing periodontal surgery. For the periodontally healthy group, biopsy samples of similar gingival tissues from periodontally healthy sites were obtained during tooth extraction either for planned orthodontic treatment or impacted third molars.

### 2.2 Epidemiological investigation on association of redondoviruses with periodontitis

The following information for each patient was collected using questionnaires: demographic and socioeconomic status (e.g., gender, age, height, weight, and education level); oral hygiene habits (e.g., tooth brushing frequency, use of dental floss and mouthwash); health behaviors (e.g., frequency of smoking and alcohol consumption), and physical condition (systemic diseases and discomfort). Clinical examinations and evaluations of caries indices (using decayed, missed, and filled teeth (DMFT); decayed, missed, and filled surfaces (DMFS)), periodontal indices, and oral mucosal diseases were then performed. Ten percent of the participants were reexamined to evaluate intra-examiner reliability. The Cohen’s kappa values (κ) for all parameters (caries and periodontal indices) were >0.8, suggesting good intra-examiner reliability.

In the present case–control study, redondoviruses were detected using nested polymerase chain reaction (PCR) assays. The QIAamp DNA Mini kit (QIAGEN, Dusseldorf, Germany) was used to extract DNA from tissue specimens following the manufacturer’s guidelines. Primers targeting the Cap region were designed corresponding to the conserved segment in redondoviruses (Supplementary Table S1). The first-round PCR was performed using a 20 μl aliquot of the reaction mixture (containing 2 μl extracted DNA, 10 μl PrimerSTAR Max Premix, and 4 pmol of primers) that was initially denatured (at 94°C; 3 min). This was followed by thirty-seven cycles: 30 s at 94°C, 30 s at 55°C, and 40 s at 72°C and a final extension at 72°C for 7 min. The second-round reaction volume (50 μl) contained 10 pmol of primers, 25 μl of PrimerSTAR Max Premix, and 1 μl of the first-round PCR product. The cycling parameters were similar to those applied in the first round. Deionized water was used as the negative control and showed no positive results during the two rounds. Electrophoresis on agarose gels (1%) was used to resolve the amplification products and for further sequencing. The detection frequency (%) of the redondovirus-positive products was analyzed.

### 2.3 Identification of the novel redondoviruses

Based on the Basic Local Alignment Search Tool (BLAST) score (E-value < 0.001), five novel viral sequences that have low homologies with known redondoviruses were further analyzed. Primers were designed for amplification (Supplementary Table S1) based on the novel viral sequences, followed by inverse nested PCR and the amplicons were subjected to Sanger sequencing. An overlapping PCR fragment of the circular genome was then obtained and sequenced for identification. The Takara LA Taq polymerase (LA PCR Kit Ver.2.1, TaKaRa, Dalian, China) was used for the amplification process as follows: at 94°C (3 min, then five cycles for 1 min each), at 60°C (1 min), and at 72°C (3.5 mins), followed by thirty cycles of denaturation (94°C; 30 s), annealing (55°C; 30 s), and extension (72°C; 3.5 mins). For the extension stage, every cycle included an additional 1 s and the final extension at 72°C for 10 min. Similar parameters were used for the first and second rounds. Electrophoresis and agarose gels (1%) were used to analyze the PCR amplicons followed by cloning and sequencing.

### 2.4 Nucleotide and amino acid sequence analysis

The computer software MegAlign (DNAStar Inc., Madison, WI, USA) and the open reading frame (ORF) Finder (http://www.ncbi.nlm.nih.gov/projects/gorf/) were used to analyze the sequences. In order to predict the putative functions of the translated proteins on the basis of ambisense ORFs, known protein sequences were compared using the GenBank database and BLASTp program. For each protein, the E-value was recorded and assigned to the best-matching reference viral protein. The ORF conserved domains were identified through NCBI’s CD-search. Protein folding predictions were performed with PHYRE2 (Kelley et al. 2015) and Forna (http://rna.tbi.univie.ac.at/forna/) (Kerpedjiev et al. 2015) using the default parameters. Motifs were detected with Multiple EM for Motif Elicitation (MEME; http://meme-suite.org) using the default parameters (Tran and Huang 2014). An online tool (http://www.cbs.dtu.dk/services/BepiPred/) with an epitope threshold of 0.5 was used to predict the epitopes for the new redondovirus Cap proteins. The I-TASSER online tool (Zhang et al. 2013) (https://zhanglab.ccmb.med.umich.edu) was used to predict the structure of the Cap proteins. The viral structures were illustrated with PyMOL (version 2.3.5) (Li et al. 2018). The PROTEAN software (DNASTAR’s Lasergene, Inc., Madison, WI, USA) was used to analyze the secondary structure of the amino acid sequences of the identified epitopes (Xu et al. 2019).

### 2.5 Phylogenetic analysis

Sequences were aligned using ClustalW included in the Molecular Evolutionary Genetics Analysis program (MEGA, version X, USA). Phylogenetic analysis was performed using the maximum likelihood (ML) method, Tamura-Nei model (the whole genome sequences), Le_Gascuel_2008 model (Cap and Rep amino acid sequences), and 1,000 bootstrap replications in MEGA X (Tamura and Nei 1993; Le and Gascuel 2008; Kumar et al. 2018; Stecher et al. 2020). Sequences with E-values <10^−5^ were regarded as homologs. The reference virus sequences had been previously identified as redondoviruses strains (Abbas et al. 2019). Four types of CRESS-DNA sequences belonging to the *Geminiviridae*, *Circoviridae*, *Smacoviridae*, and *Genomoviridae* families according to the International Committee on Taxonomy of Viruses (ICTV) were retrieved from NCBI. The phylogenetic trees were visualized using iTOL (Version 5.5.1, https://itol.embl.de) (Letunic and Bork 2019). In addition, MEGA X was used to perform homology analysis. Recombination analysis of the redondovirus genomes was performed using the RDP3 (Heath et al. 2006) and SimPlot programs (Baumgarte et al. 2008).

### 2.6 Statistical analysis

The data were statistically analyzed using SPSS software (version 20.0; IBM Corporation, Armonk, NY, USA) with the significance level set at *P *<* *0.05. Descriptive statistics were employed for the participants’ socio-demographic characteristics and periodontal measurements. The differences in the gender distribution and lifestyle factors and the prevalence of redondoviruses were evaluated using χ^2^ tests. The Student’s *t* test was applied to compare the differences in the caries indices (DMFT and DMFS) between the groups. Logistic regression analysis was used to calculate the odds ratio (OR) and to explore factors associated with chronic periodontitis. Variables with *P* values <0.5 in bivariate analyses were entered into the regression model.

## 3. Results

### 3.1 Association analysis of redondoviruses and periodontitis

There were no significant differences in age or gender between the chronic periodontitis and healthy groups (*P *=* *0.137 and 0.439, respectively, Table 1). The mean values for PD, CAL, PLI, and GI in the periodontitis group were 6.34 mm, 3.68 mm, 2.52, and 2.37, respectively, and the corresponding values in the healthy group were 1.36 mm, 0.08 mm, 0.44, and 0.17, respectively. In terms of the associations between periodontitis and physical or lifestyle factors, a lower tooth brushing frequency was associated with chronic periodontitis (*P *=* *0.037, Supplementary Table S2).

**Table 1.** Comparison of the characteristics of periodontitis and control groups participants.

Group	Periodontitis group	Control group	*P*
Age (mean±SD)	41.38 ± 12.70	39.17 ± 10.18	0.137
Gender (N, %)			0.439
Male	56 (46.67%)	62 (51.67%)	
Female	64 (53.33%)	58 (48.33%)	
Redondoviruses (N, %)			0.001
Positive	86 (71.67%)	62 (51.67%)	
Negative	34 (28.33%)	58 (48.33%)	

SD, standard deviation.

The PCR results for redondovirus detection were positive for 86 (71.67%) of the periodontitis patients and sixty two (51.67%) of the healthy group participants (*P *=* *0.001), suggesting an association between redondoviruses and chronic periodontitis. To explore the factors associated with chronic periodontitis after controlling various confounding factors, multiple logistic regression analysis was performed (Table 2). Healthy periodontium and periodontitis were dichotomized as 0 and 1, respectively. The regression model indicated a significant association between chronic periodontitis and redondovirus infection, with an adjusted OR of 2.53. In addition, chronic periodontitis was associated with profound work stresses, low tooth brushing frequency, and low educational level (Table 2).

**Table 2.** Multiple logistic regression model for chronic periodontitis (0: periodontal healthy, 1: periodontitis).

	Odds ratio	95% CI	*P*
Redondovirus infection			0.003
Negative^a^			
Positive	2.53	1.38–4.62	
Tooth brushing			0.006
≥2×/day^a^			
1×/day	2.53	0.32–20.23	
Never or seldom	2.95	1.50–5.79	
Work pressure (N, %)			0.001
Heavy	2.37	1.12–5.04	
Medium	0.52	0.25–1.07	
Low^a^			
Educational level			0.027
College and below	2.68	1.21–5.96	
Undergraduate	2.61	1.21–5.62	
Master's degree and above^a^			

a Reference group.

CI, confidence interval.

### 3.2 Discovery of novel redondoviruses

Inverse nested PCR and Sanger sequencing of novel viral sequences from the periodontitis gingival specimens revealed five complete circular virus genomes. According to the ICTV (Van 2016; Van Regenmortel 2019) and the recent *Redondoviridae* study (Abbas et al. 2019), the present study results suggested the five new virus sequences may be novel vientoviruses. The five source patients (three men and two women; aged 35–55 years) from whom these novel viruses were identified exhibited BOP (+) and alveolar bone resorption and their mean values for PD, CAL, PLI, and GI of the teeth were 10.20 mm, 5.20 mm, 1.60, and 2.40, respectively.

Based on the best BLAST results (E-value <0.001), the new virus sequences had the highest identity with reported viruses in the *Redondoviridae* family, a new group of CRESS DNA viruses. The viral genomes identified as redondoviruses were deposited into GenBank (accession numbers: MT482428MT482432) and were named ‘human periodontal circular like virus (HPeCV)’ with the corresponding source numbers (HPeCV-1, -10, -11, -25, -26, respectively).

### 3.3 Novel redondovirus genome organization

**Figure 1.**
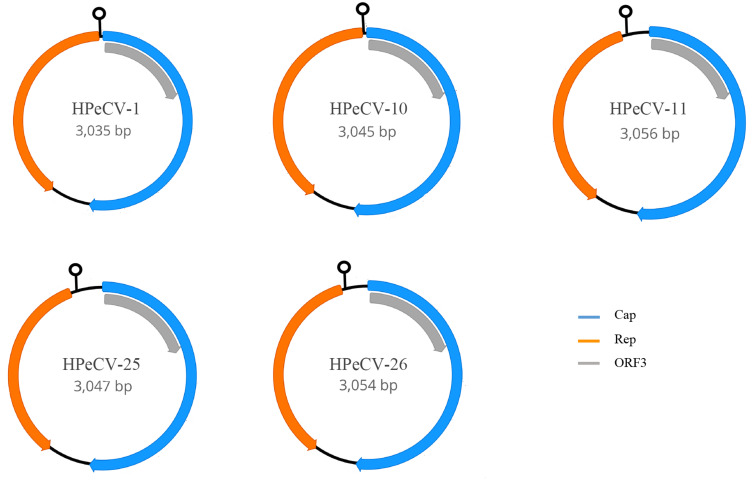
The genome organization of the novel HPeCVs. The genomic architecture contains three ambisense ORFs encoding the Cap protein, a Rep protein, and ORF3. A predicted stem-loop structure is located at the 5′ end of Rep.

**Figure 2.**
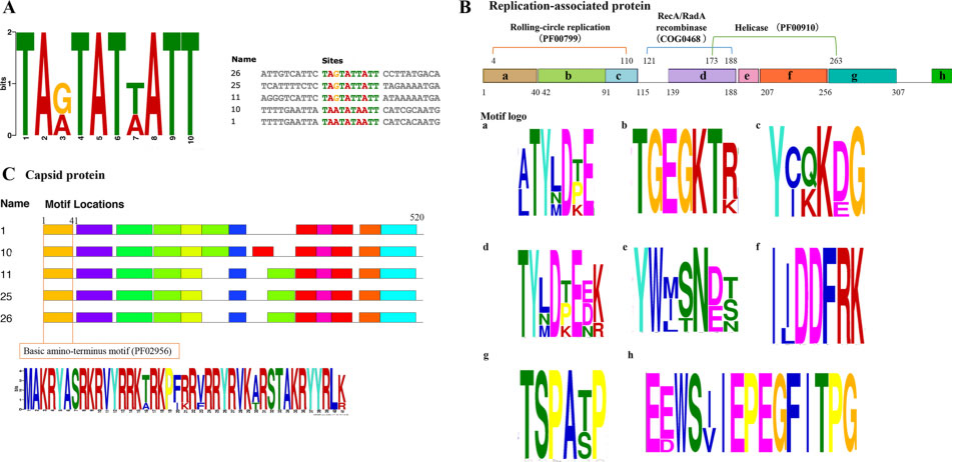
Putative conserved motifs in HPeCVs. The height of each motif letter represents the frequency. (A) The predicted motif for the replication origin in the stem-loop structure. (B) The predicted conserved motifs in the Rep protein contain the initiator protein in a rolling circle replication, helicase, and RecA/RadA recombinase. The positions for the motifs are given using HPeCV-26 sequence. (C) Predicted conserved motifs in Cap protein.

The length of the newly identified circular viral genomes was 3,035–3,056 bp. Further analysis of the genomic organization of the five novel viral sequences showed three major ORFs encoding a Cap protein (500–531 amino acid protein), Rep protein (346–396 amino acid protein), and ORF3 (200 amino acid protein) (Fig. 1). ORF3 overlaps with the Cap sequence; however, the function is not clear as it is not aligned with any reported proteins. Similar to other CRESS-DNA virus families (Krupovic et al. 2016; Breitbart et al. 2017; Varsani and Krupovic 2018), the putative ORFs for redondoviruses are separated by small and large intergenic regions. The redondovirus genomes comprised a conserved motif (‘TANTATNATT’) inside the stem-loop structure, followed by short direct repeats at the 5′ end of Rep in a small intergenic region, which may be the origin of replication (Fig. 2A). These findings are in agreement with the arrangement of redondovirus strains (Abbas et al. 2019).

The predicted Rep proteins (Fig. 2B) contain two conserved domain-containing proteins: a replication initiator protein associated with rolling-circle replication (Gemini_AL1; Pfam: PF00799) and a helicase domain within the P-loop NTPase superfamily (Pfam: PF00910). A third conserved motif, RecA/RadA recombinase from the cl33895 superfamily, was also observed in the present study. This motif is involved in replication, recombination, and repair (COG0468). The putative Cap proteins contain a conserved basic amino-terminus motif (Pfam: PF02956) (Fig. 2C). The detailed motifs for Cap are shown in Supplementary Fig. S1.

### 3.4 Phylogenetic and recombination analysis of HPeCVs

**Figure 3.**
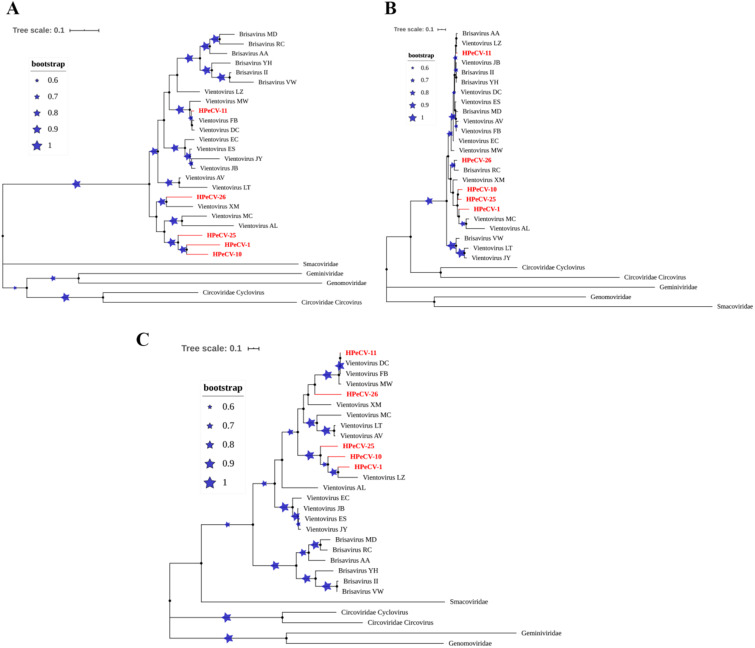
Phylogenetic trees of novel HPeCVs formed using the ML method and 1,000 bootstrap replicates. The blue stars with branches represent the bootstrap values. The novel HPeCVs are colored red. (A) Complete nucleotide sequences, using Tamura-Nei model; (B) Capsid protein sequences, using Le_Gascuel_2008 model; (C) Rep protein sequences, and Le_Gascuel_2008 model.

Phylogenetic trees and pairwise identities were constructed using the whole genome sequences, the amino acid sequences of Cap and Rep for the five HPeCVs, and the corresponding reference sequences from the *Redondoviridae* and other CRESS virus families (Fig. 3). The phylogenetic alignment of the sequences predicted using the programs ClustalX and MegAlign demonstrated a closer evolutionary relationship between HPeCVs and vientoviruses in the whole genome tree. In addition, a visible cluster consisting of HPeCV-1, HPeCV-10, and HPeCV-25 was evident in the phylogenetic tree. Sequence comparison revealed that the novel HPeCVs had 73.4 per cent to 98.3 per cent nucleotide identities with other redondoviruses (Supplementary Table S3).

The Rep protein tree suggests that the novel HPeCVs are also closely related to the Vientovirus genus, which belongs to the *Redondoviridae* family. The Rep proteins for HPeCV-1, HPeCV-10, and HPeCV-25 formed a cluster that showed the highest amino acid sequence identity with Vientovirus_LZ (GenBank accession No. MK059769) (76.9%, 69.6%, 64.1%, respectively). HPeCV-11 shares 99.7 per cent identity with Vientovirus_FB (GenBank accession No. MK059763), while HPeCV-26 shares 66.8 per cent identity with Vientovirus_XM (GenBank accession No. MK059771). The nucleotide identities between different redondoviruses for the Rep protein are presented in Supplementary Table S4.

The Cap proteins within the *Redondoviridae* family share a level of identity ranging from 70.4 per cent to 97.4 per cent, which is remarkably higher than the degree of identity among other CRESS virus families (≤26.0%) (Supplementary Table S5). A comparison of the Cap amino acid sequences revealed that strains from the two genera (Vientovirus and Brisavirus) were cross-clustered in the tree, indicating discordant relationships from those of Rep and the complete sequence trees and suggesting that recombination may exist in redondoviruses.

**Figure 4.**
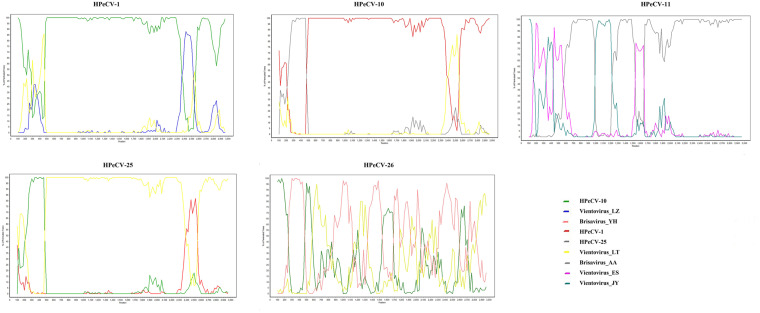
Recombination analysis of the novel HPeCVs by Bootscan analysis. Bootstrapped phylogenesis is performed in a sliding window.

To identify recombination events in the novel HPeCVs, recombination analysis was conducted with the complete genomes (Fig. 4). HPeCV-1 may be a recombinant resulting from recombination events that occur between HPeCV-10 and Vientovirus LZ and HPeCV-25 may be a recombinant mainly from HPeCV-1, HPeCV-10, and Vientovirus LT. Notably, recombination can exist in both the Cap and Rep sequences in the genome.

### 3.5 Predicted immune epitopes of HPeCVs

**Figure 5.**
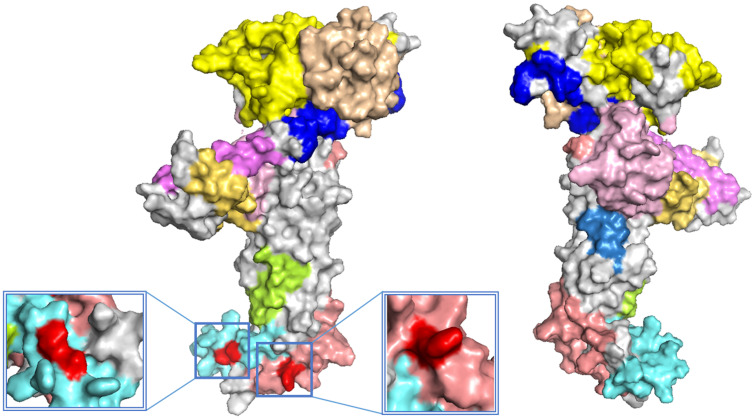
Prediction of immune epitopes of the novel HPeCV-26. Various predicted epitopes in the Cap protein are indicated by different colors. Amino acid sites 14 (Arg, epitope probability = 0.58), 56 (Ala, epitope probability = 0.53), and 59 (Gln, epitope probability = 0.58) are indicated in red (C-score = −1.60). The confidence for this model was quantitatively measured using the C-score, which was calculated based on the significance of threading template alignments and the converged parameters in the structure assembly simulations. The C-score is typically in the range of [-5, 2] and a higher value indicates a model with a higher confidence and vice versa.

The Cap protein structure model was constructed and its antigenic epitope was predicted. Potential epitopes located on the surface of the Cap protein were indicated using various colors in the predicted model (Fig. 5 and Supplementary Fig. S2). The epitopes bridged the majority of the protein surface. However, combining epitope prediction with amino acid analysis using PROTEAN revealed that the epitope regions with the highest antigenic index and hydrophilicity in each Cap protein were similar in all five newly identified strains. For example, HPeCV-26 contained epitopes at amino acids sites 14 (Arg), 56 (Ala), and 59 (Gln) in the Cap protein sequence and showed the highest antigenic index, surface probability, and hydrophilicity (Fig. 5 and Supplementary Fig. S3).

## 4. Discussion

The relationship between recently identified redondoviruses and chronic periodontitis was investigated in the present case–control study. Five novel viruses belonging to the new *Redondoviridae* viral family were identified from gingival tissue biopsies of chronic periodontitis patients. Considering the diversity of redondoviruses, the complete circular genomes, phylogenetic relationship, and biological characteristics of the identified redondoviruses were further investigated.

The present study revealed a significantly higher prevalence of redondovirus infection (71.67%) among chronic periodontitis patients compared to that in healthy individuals (51.67%, *P* = 0.001). The regression model showed that chronic periodontitis was significantly associated with redondovirus infection (adjusted OR of 2.53). A previous study indicated that oral redondoviruses mainly belong to the Vientovirus genus (Abbas et al. 2019). Based on the conserved fragments in vientoviruses, primers for epidemiological investigation were designed to detect the viruses and identify the new strains. Although viruses are abundant in biological systems, the majority of viromes mutate their genetic elements rapidly and therefore, remain unclassified and uncharacterized (Aggarwala et al. 2017; Krishnamurthy and Wang 2017). In recent years, a number of newly identified virus families have provided a new perspective to understand the pathogenesis of various diseases including periodontal diseases and have been explored extensively (Abeles et al. 2014; Ly et al. 2014; Pinto et al. 2016). For example, various organisms such as the Epstein–Barr virus (EBV), herpes simplex virus-1 (HSV-1), and human cytomegalovirus (HCMV) have been reported to contribute to the pathogenesis of periodontal diseases (Bilder et al. 2013; Das et al. 2012; Slots, 2010). However, the association between the virome composition and periodontal diseases is not yet fully understood. We previously investigated the association of novel bacteriophages and anelloviruses with periodontitis (Zhang et al. 2016; Zhang et al. 2017; Zhang et al. 2019). Viruses may contribute significantly to the pathogenesis of periodontitis by subverting various molecular signaling pathways in inflammatory immune cells (Meyle and Chapple 2015). Recently, Abbas et al. reported that redondoviruses were the second most abundant DNA viruses in the human virome and demonstrated a similar prevalence but higher genome quantities in cohorts composed of critically ill and healthy individuals (Abbas et al. 2019). Additionally, the presence of redondoviruses was reported in oro-respiratory tissues and may increase in various disorders including periodontitis (Abbas et al. 2019). Our findings are in agreement and suggested a high frequency of redondoviruses in the inflamed periodontal tissues of Chinese residents. These findings suggest that redondoviruses are involved in the pathogenesis of periodontitis. However, the relationship between this recently discovered virus family and periodontal disease is not fully understood and is debatable, especially in cases of co-infection with other viruses such as HSV-1, HCMV, and EBV. The epidemic situation among other races and whether the redondovirus is related to ethnicity also require further investigations.

Five new redondoviruses (HPeCVs) belonging to the Vientovirus genus were identified and genetically analyzed in the present study. Analysis using ML phylogenetic trees for the complete genome as well as the Rep and Cap protein sequences revealed the phylogenetic relationships between viral proteins in *Redondoviridae* and other CRESS virus families. The present study showed that the protein and genome organizations of redondoviruses are more similar to each other than to other CRESS family members (Fig. 2). A visible cluster consisting of HPeCV-1, HPeCV-10, and HPeCV-25 was observed in the complete genome and Rep protein phylogenetic trees. We propose that these three viruses belong to the new group of redondoviruses. Intriguingly, the three phylogenetic trees are not congruent with each other. In the Cap protein tree, strains from the two genera (Vientovirus and Brisavirus) are cross clustered, corresponding to the intra-familial recombination of various genomes and leading to chimeric entities encoding Rep and Cap with variable evolutionary histories. Similar findings in other CRESS families have been reported (Lefeuvre et al. 2009; Varsani and Krupovic 2017, 2018). Recombination analysis provided evidence that recombination events may be common in *Redondoviridae*. We suggest that the relatively conserved Rep protein can be investigated for further understanding of the origin of redondoviruses. In addition, we analyzed the putative epitopes that may be exposed on the surface of the Cap protein, which are associated with the host immune response. Amino acid sites 14 (Arg), 56 (Ala), and 59 (Gln) on the Cap protein sequence of HPeCV-26 were predicted to be potential immune epitopes. The sites were relatively conserved among the strains reported in this study. Further experimental studies are required to verify the immunogenicity of the predicted sites.

Although the present study expands the current understanding of the genetic diversity and pathogenicity of *Redondoviridae*, there are a few limitations and corresponding future research directions. First, the epidemiological analysis results were limited to vientoviruses. Strain-specific disease association analysis can further strengthen the evidence for periodontitis-related redondoviruses and their pathogenicity in periodontitis. Second, since recombination may exist in Cap sequences in these genomes, additional studies to determine whether recombination events can change the immunogenicity of the viruses and their transmission patterns are needed. Third, although *Redondoviridae* currently includes only two representative genera, more new members are expected to be added to the family in the near future. The worldwide epidemiology and more detailed genotyping are probably necessary to describe the true diversity of this new family. Fourthly, reports confirming replication of redondoviruses in human cells or of sero-conversion to these viruses are still lacking. Therefore, it is also conceivable that the existence of redondovirus genomes in gingival tissues may result from contamination of oral cellular organisms with release of their viruses into oral cavity. Identifying these viruses' host will help researchers better understand the role of redondoviruses in periodontitis.

In conclusion, we observed a high prevalence of redondoviruses in the periodontal tissues of Chinese residents in a small cohort. Chronic periodontitis patients showed a significantly higher prevalence of the Vientovirus genus than healthy controls. Furthermore, five novel redondoviruses (namely HPeCVs) were identified from the periodontal tissues evaluated in the current study and were further analyzed to determine their genomic structures and phylogenies. The genetic characterization enhanced the current understanding of the genetic diversity and pathogenicity of redondoviruses as well as their association with chronic periodontitis in humans.

## Data availability

The complete genome sequences for the HPeCVs determined in this study are available in GenBank under the accession numbers MT482428∼MT482432.

## Supplementary Material

veab033_Supp
