# Supplementary material for: Redondoviridae and periodontitis: a case–control study and identification of five novel redondoviruses from periodontal tissues
Source: Virus Evol. 2021 Apr 12;7(1):veab033. doi: 10.1093/ve/veab033 (PMC8088815; doi:10.1093/ve/veab033)
Supplement: veab033_Supp [file veab033_Supp.zip › veab033_Supplementary_Data.docx]

***Redondoviridae* and periodontitis: A case-control study and identification of five novel redondoviruses from periodontal tissues**

**Yu Zhang^1,3,4,*^, Chunmei Wang^2,*^, Xiping Feng^1,3,4^, Xi Chen^1,3,4,#^ , Wen Zhang^5,#^**

^1^Department of Preventive Dentistry, Shanghai Ninth People's Hospital, College of Stomatology, Shanghai Jiao Tong University School of Medicine, Shanghai, PR China;

^2^Shanghai Veterinary Research Institute, Chinese Academy of Agricultural Sciences, Shanghai, PR China;

^3^National Clinical Research Center for Oral Diseases, Shanghai, PR China;

^4^Shanghai Key Laboratory of Stomatology & Shanghai Research Institute of Stomatology, Shanghai, PR China;

^5^School of Medicine, Jiangsu University, Zhenjiang, PR China.

**^*^**These authors contributed equally to this study and share first authorship.

^#^ these authors contributed equally to this study and share corresponding authorship.

**Corresponding author:**

Wen Zhang (zhangwen@ujs.edu.cn), School of Medicine, Jiangsu University, 3 Yizheng Road, Zhenjiang, PR China.

Xi Chen (chenxi9h@126.com), Department of Preventive Dentistry, Shanghai Ninth People's Hospital, College of Stomatology, Shanghai Jiao Tong University School of Medicine, 639 Zhizaoju Road, Shanghai, PR China.

**Supplemental Tables**

Table S1 Primers for PCR

|  | Upstream Primer（5’ to 3’） | Downstream Primer（5’ to 3’） |
| --- | --- | --- |
| Epidemiological round 1 | G*G*CTTAAGAGGGCTGCTAGG | A*G*GTAACCGGCGTAAGTATG |
| Epidemiological round 2 | GTCAAGAAGATTAGAAGGGC | AGCCATACAAATAAGGAGG |
| #1 round 1 | C*T*TACTGAACAAGAAATGGC | A*C*AAACCATAGAAGCAGGTC |
| #1 round 2 | TTTCTTTACAAAATGGGTGC | AAGGCAACTGAATAAATTCC |
| #10 round 1 | G*A*ACAGATGGATACTTTTGC | A*C*AAACCATAGAAGCAAGTC |
| #10 round 2 | AAGTTCCTTTACAAAATGGG | AGGCAATTGAATAAATTCTG |
| #11 round 1 | A*C*TCCAGTTGGTTACCCTAT | A*C*AAACCATAGAAGCAAGTC |
| #11 round 2 | TTTCTTTACAAACTGGGTGC | AGGCAATTGAATAAATTCTG |
| #25 round 1 | A*T*CCTCTTAATGAGGACCAG | C*T*ACAAACCAAAGAAGCAAG |
| #25 round 2 | AAGTTCCTTTACAAAATGGG | AGGCAATTGAATAAATTCTG |
| #26 round 1 | A*C*TGCAACTGATATGCGACA | G*C*AAACCATAGAAGCAAGTC |
| #26 round 2 | GTGAACTTTATACGTCCAGG | AGGCAATTGAATAAATTCTG |

**Table S2.** Physical and lifestyle characteristics of the chronic periodontitis and control group participants

| Group | Periodontitis group | | Control group | | *P* |
| --- | --- | --- | --- | --- | --- |
| DMFT (mean±SD) | 1.38±1.50 | 1.02±1.35 | | 0.202 | |
| DMFS (mean±SD) | 1.90±2.14 | 1.74±2.27 | | 0.118 | |
| Place of birth (N, %) |  |  | | 0.398 | |
| Shanghai region | 81 (67.5%) | 87 (72.5%) | |  | |
| Other | 39 (32.5%) | 33 (27.5%) | |  | |
| BMI (kg/m^2^, N, %) |  |  | | 0.227 | |
| <18 | 4 (3.3%) | 9 (7.5%) | |  | |
| 18-24 | 68 (56.7%) | 72 (60.0%) | |  | |
| >24 | 48 (40.0%) | 39 (32.5%) | |  | |
| Monthly income, RMB (N, %)^†^ |  |  | | 0.076 | |
| ≤3000 | 3 (2.7%) | 0 (0.0%) | |  | |
| 3001–6000 | 28 (24.8%) | 25 (21.7%) | |  | |
| 6001–12 000 | 43 (38.1%) | 60 (52.2%) | |  | |
| >12 000 | 39 (34.5%) | 30 (26.1%) | |  | |
| Educational level (N, %) |  |  | | 0.161 | |
| College and below | 42 (35.0%) | 37 (30.8%) | |  | |
| Undergraduate | 53 (44.2%) | 45 (37.5%) | |  | |
| Master's degree and above | 25 (20.8%) | 38 (31.7%) | |  | |
| Tooth brushing (N, %) |  |  | | 0.037 | |
| ≥2×/day | 74 (61.7%) | 92 (76.7%) | |  | |
| 1×/day | 44 (36.7%) | 26 (21.7%) | |  | |
| Never or seldom | 2 (1.7%) | 2 (1.7%) | |  | |
| Dental floss (N, %)^†^ |  |  | | 0.101 | |
| Yes | 26 (21.7%) | 16 (13.6%) | |  | |
| Never or seldom | 94 (78.3%) | 102 (86.4%) | |  | |
| Mouthwash use (N, %) |  |  | | 0.743 | |
| Yes | 24 (20.0%) | 22 (18.3%) | |  | |
| Never or seldom | 96 (80.0%) | 98 (81.7%) | |  | |
| Work pressure (N, %) |  |  | | 0.059 | |
| Heavy | 46 (38.3%) | 29 (24.2%) | |  | |
| Medium | 41 (34.2%) | 52 (43.3%) | |  | |
| Low | 33 (27.5%) | 39 (32.5%) | |  | |
| Physical activity (N, %)^†^ |  |  | | 0.812 | |
| Yes | 48 (40.7%) | 47 (39.2%) | |  | |
| No | 70 (59.3%) | 73 (60.8%) | |  | |
| Smoking (N, %) |  |  | | 0.552 | |
| Yes | 16 (13.3%) | 13 (10.8%) | |  | |
| No | 104 (86.7%) | 105 (89.2%) | |  | |
| Drinking (N, %) |  |  | | 0.869 | |
| Yes | 23 (19.2%) | 22 (18.3%) | |  | |
| No | 97 (80.8%) | 98 (81.7%) | |  | |

†Some data are missing for these variables.

SD: standard deviation

BMI: body mass index; DMFT: decayed, missing, and filled tooth; DMFS: decayed, missing, and filled surface; N: Number of participants examined; RMB, yuan, renminbi (currency in China).

**Table S3.** Sequence identity of complete genome of novel HPeCVs with other reference strains.

|  | HPeCV-1 | HPeCV-10 | HPeCV-11 | HPeCV-25 | HPeCV-26 |
| --- | --- | --- | --- | --- | --- |
| HPeCV-1 |  |  |  |  |  |
| HPeCV-10 | 0.84 |  |  |  |  |
| HPeCV-11 | 0.78 | 0.79 |  |  |  |
| HPeCV-25 | 0.82 | 0.85 | 0.79 |  |  |
| HPeCV-26 | 0.78 | 0.80 | 0.82 | 0.80 |  |
| Geminiviridae | 0.33 | 0.33 | 0.33 | 0.33 | 0.34 |
| Genomoviridae | 0.34 | 0.34 | 0.34 | 0.34 | 0.32 |
| Circoviridae_Cyclovirus | 0.40 | 0.42 | 0.42 | 0.41 | 0.42 |
| Circoviridae_Circovirus | 0.32 | 0.32 | 0.33 | 0.33 | 0.33 |
| Smacoviridae | 0.34 | 0.34 | 0.33 | 0.34 | 0.33 |
| Brisavirus_AA | 0.74 | 0.75 | 0.81 | 0.75 | 0.77 |
| Brisavirus_II | 0.73 | 0.75 | 0.82 | 0.75 | 0.78 |
| Brisavirus_MD | 0.75 | 0.76 | 0.82 | 0.76 | 0.78 |
| Vientovirus_MW | 0.79 | 0.80 | 0.95 | 0.80 | 0.81 |
| Vientovirus_XM | 0.79 | 0.81 | 0.82 | 0.81 | 0.85 |
| Vientovirus_MC | 0.79 | 0.80 | 0.77 | 0.81 | 0.79 |
| Vientovirus_LZ | 0.82 | 0.82 | 0.86 | 0.80 | 0.80 |
| Vientovirus_EC | 0.77 | 0.78 | 0.84 | 0.78 | 0.82 |
| Vientovirus_AV | 0.78 | 0.79 | 0.85 | 0.79 | 0.82 |
| Vientovirus_LT | 0.76 | 0.76 | 0.79 | 0.75 | 0.77 |
| Vientovirus_JY | 0.74 | 0.75 | 0.78 | 0.74 | 0.76 |
| Vientovirus_JB | 0.76 | 0.78 | 0.85 | 0.78 | 0.81 |
| Vientovirus_FB | 0.78 | 0.80 | 0.98 | 0.79 | 0.82 |
| Vientovirus_ES | 0.77 | 0.78 | 0.85 | 0.78 | 0.82 |
| Vientovirus_DC | 0.78 | 0.79 | 0.98 | 0.79 | 0.82 |
| Vientovirus_AL | 0.74 | 0.74 | 0.74 | 0.74 | 0.76 |
| Brisavirus_VW | 0.72 | 0.74 | 0.76 | 0.74 | 0.76 |
| Brisavirus_YH | 0.74 | 0.75 | 0.82 | 0.75 | 0.78 |
| Brisavirus_RC | 0.75 | 0.77 | 0.77 | 0.78 | 0.80 |

**Table S4.** Sequence identity of novel HPeCV Rep sequences with other reference strains.

|  | HPeCV-1 | HPeCV-10 | HPeCV-11 | HPeCV-25 | HPeCV-26 |
| --- | --- | --- | --- | --- | --- |
| HPeCV-1 |  |  |  |  |  |
| HPeCV-10 | 0.73 |  |  |  |  |
| HPeCV-11 | 0.61 | 0.59 |  |  |  |
| HPeCV-25 | 0.70 | 0.72 | 0.59 |  |  |
| HPeCV-26 | 0.57 | 0.52 | 0.66 | 0.53 |  |
| Geminiviridae | 0.12 | 0.12 | 0.12 | 0.11 | 0.12 |
| Genomoviridae | 0.16 | 0.12 | 0.13 | 0.15 | 0.15 |
| Circoviridae_Cyclovirus | 0.20 | 0.21 | 0.21 | 0.22 | 0.23 |
| Circoviridae_Circovirus | 0.19 | 0.21 | 0.19 | 0.20 | 0.18 |
| Smacoviridae | 0.17 | 0.16 | 0.16 | 0.16 | 0.13 |
| Brisavirus_AA | 0.42 | 0.40 | 0.39 | 0.42 | 0.42 |
| Brisavirus_II | 0.42 | 0.42 | 0.43 | 0.45 | 0.45 |
| Brisavirus_MD | 0.44 | 0.42 | 0.42 | 0.45 | 0.42 |
| Brisavirus_RC | 0.44 | 0.42 | 0.42 | 0.45 | 0.42 |
| Brisavirus_YH | 0.41 | 0.39 | 0.41 | 0.43 | 0.44 |
| Brisavirus_VW | 0.42 | 0.42 | 0.43 | 0.45 | 0.45 |
| Vientovirus_AL | 0.55 | 0.52 | 0.56 | 0.53 | 0.61 |
| Vientovirus_DC | 0.61 | 0.59 | 1.00 | 0.59 | 0.66 |
| Vientovirus_ES | 0.58 | 0.55 | 0.58 | 0.57 | 0.59 |
| Vientovirus_FB | 0.61 | 0.59 | 0.99 | 0.59 | 0.66 |
| Vientovirus_JB | 0.58 | 0.55 | 0.58 | 0.57 | 0.59 |
| Vientovirus_JY | 0.58 | 0.54 | 0.56 | 0.56 | 0.60 |
| Vientovirus_LT | 0.58 | 0.58 | 0.63 | 0.57 | 0.63 |
| Vientovirus_AV | 0.58 | 0.58 | 0.62 | 0.58 | 0.62 |
| Vientovirus_EC | 0.56 | 0.53 | 0.55 | 0.55 | 0.58 |
| Vientovirus_LZ | 0.77 | 0.70 | 0.58 | 0.64 | 0.55 |
| Vientovirus_MC | 0.57 | 0.56 | 0.60 | 0.56 | 0.59 |
| Vientovirus_XM | 0.59 | 0.56 | 0.66 | 0.60 | 0.67 |
| Vientovirus_MW | 0.61 | 0.59 | 0.97 | 0.59 | 0.66 |

**Table S5.** Sequence identity of novel HPeCV Cap sequences with other reference strains.

|  | HPeCV-1 | HPeCV-10 | HPeCV-11 | HPeCV-25 | HPeCV-26 |
| --- | --- | --- | --- | --- | --- |
| HPeCV-1 |  |  |  |  |  |
| HPeCV-10 | 0.85 |  |  |  |  |
| HPeCV-11 | 0.77 | 0.81 |  |  |  |
| HPeCV-25 | 0.85 | 0.91 | 0.81 |  |  |
| HPeCV-26 | 0.78 | 0.84 | 0.85 | 0.83 |  |
| Geminiviridae | 0.13 | 0.13 | 0.14 | 0.12 | 0.13 |
| Genomoviridae | 0.16 | 0.15 | 0.15 | 0.16 | 0.15 |
| Circoviridae_Cyclovirus | 0.25 | 0.26 | 0.25 | 0.23 | 0.26 |
| Circoviridae_Circovirus | 0.14 | 0.15 | 0.16 | 0.16 | 0.18 |
| Smacoviridae | 0.09 | 0.11 | 0.10 | 0.10 | 0.09 |
| Brisavirus_AA | 0.77 | 0.82 | 0.96 | 0.82 | 0.85 |
| Brisavirus_II | 0.77 | 0.82 | 0.97 | 0.81 | 0.85 |
| Brisavirus_MD | 0.74 | 0.79 | 0.95 | 0.78 | 0.83 |
| Vientovirus_MW | 0.81 | 0.87 | 0.91 | 0.87 | 0.87 |
| Vientovirus_XM | 0.80 | 0.87 | 0.84 | 0.87 | 0.88 |
| Vientovirus_MC | 0.79 | 0.84 | 0.75 | 0.86 | 0.77 |
| Vientovirus_LZ | 0.77 | 0.82 | 0.98 | 0.81 | 0.85 |
| Vientovirus_EC | 0.78 | 0.83 | 0.96 | 0.83 | 0.87 |
| Vientovirus_AV | 0.78 | 0.83 | 0.93 | 0.82 | 0.86 |
| Vientovirus_LT | 0.72 | 0.74 | 0.78 | 0.72 | 0.74 |
| Vientovirus_JY | 0.72 | 0.73 | 0.78 | 0.71 | 0.74 |
| Vientovirus_JB | 0.77 | 0.82 | 0.97 | 0.81 | 0.85 |
| Vientovirus_FB | 0.77 | 0.82 | 0.95 | 0.82 | 0.86 |
| Vientovirus_ES | 0.77 | 0.82 | 0.94 | 0.81 | 0.85 |
| Vientovirus_DC | 0.77 | 0.83 | 0.96 | 0.82 | 0.86 |
| Vientovirus_AL | 0.71 | 0.72 | 0.70 | 0.70 | 0.71 |
| Brisavirus_VW | 0.73 | 0.78 | 0.81 | 0.76 | 0.81 |
| Brisavirus_YH | 0.77 | 0.82 | 0.97 | 0.81 | 0.85 |
| Brisavirus_RC | 0.78 | 0.84 | 0.84 | 0.87 | 0.92 |

**Supplemental Figures**


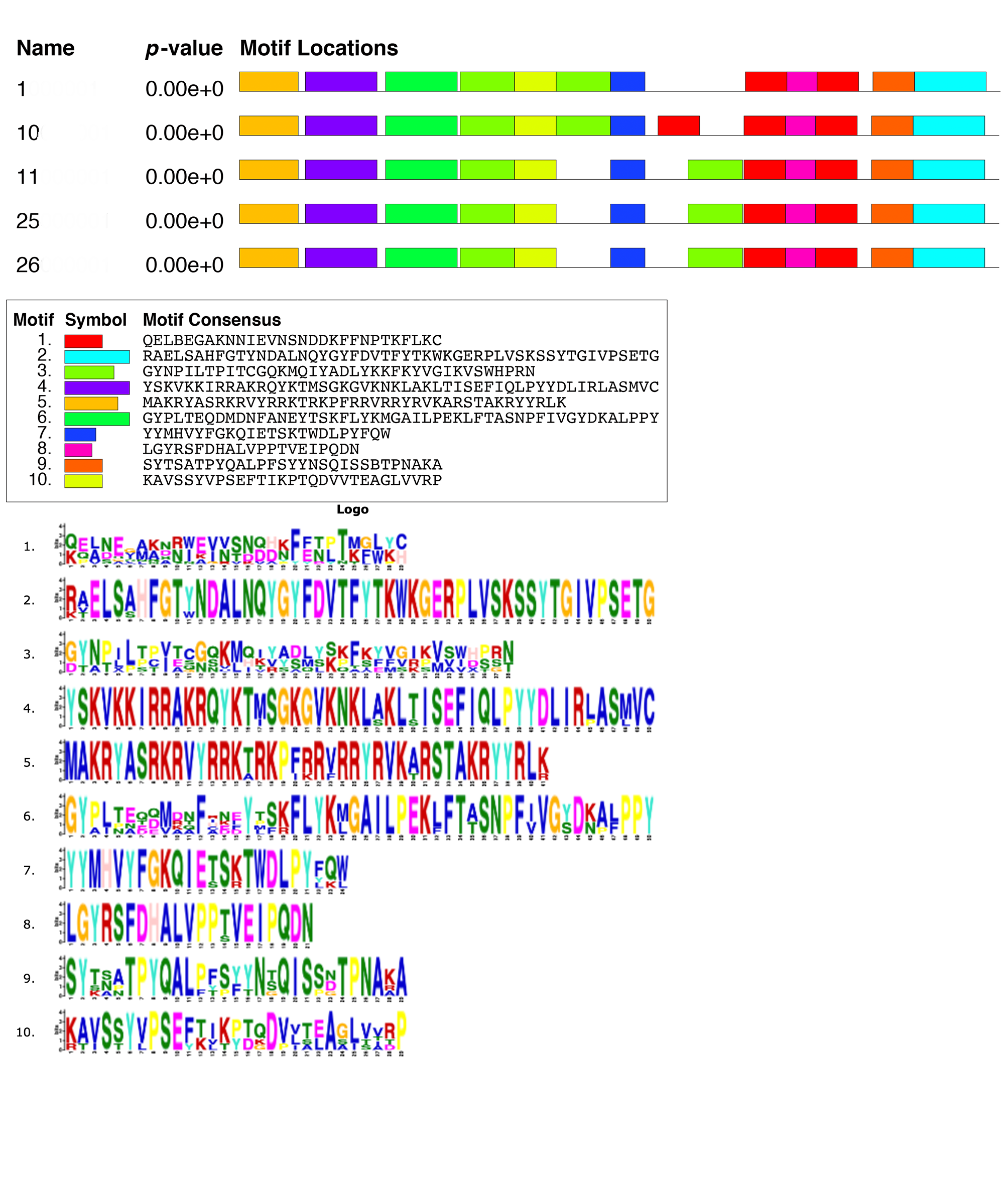


Figure S1. Putative conserved motifs in HPeCV Cap proteins.


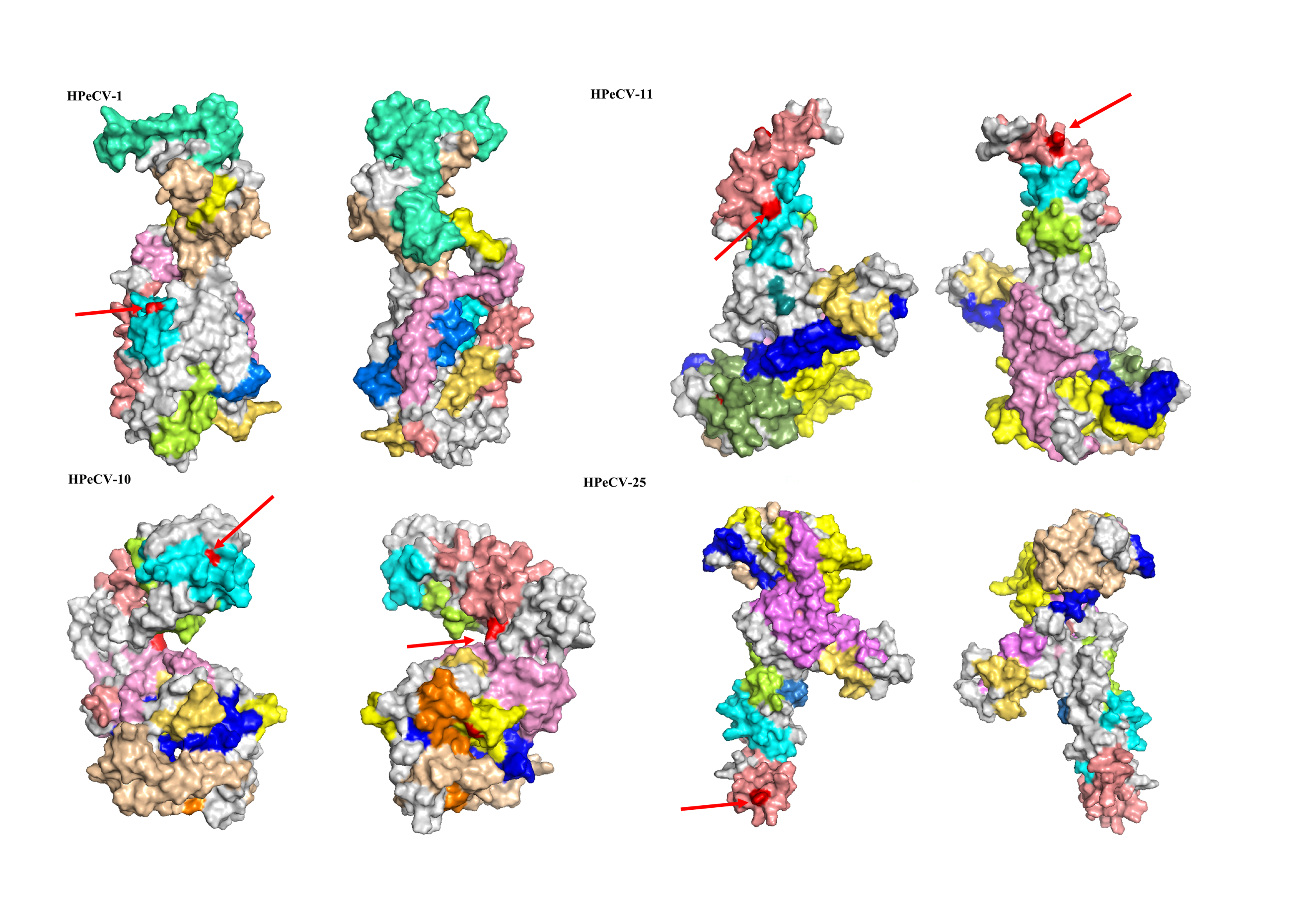


Figure S2. Prediction of immune epitopes for the novel HPeCVs. The various predicted epitopes in the Cap protein are indicated by different colors. For HPeCV-1, HPeCV-10, HPeCV-11, and HPeCV-25, the C-scores were -1.67, -2.40, -1.51, and -1.55, respectively.


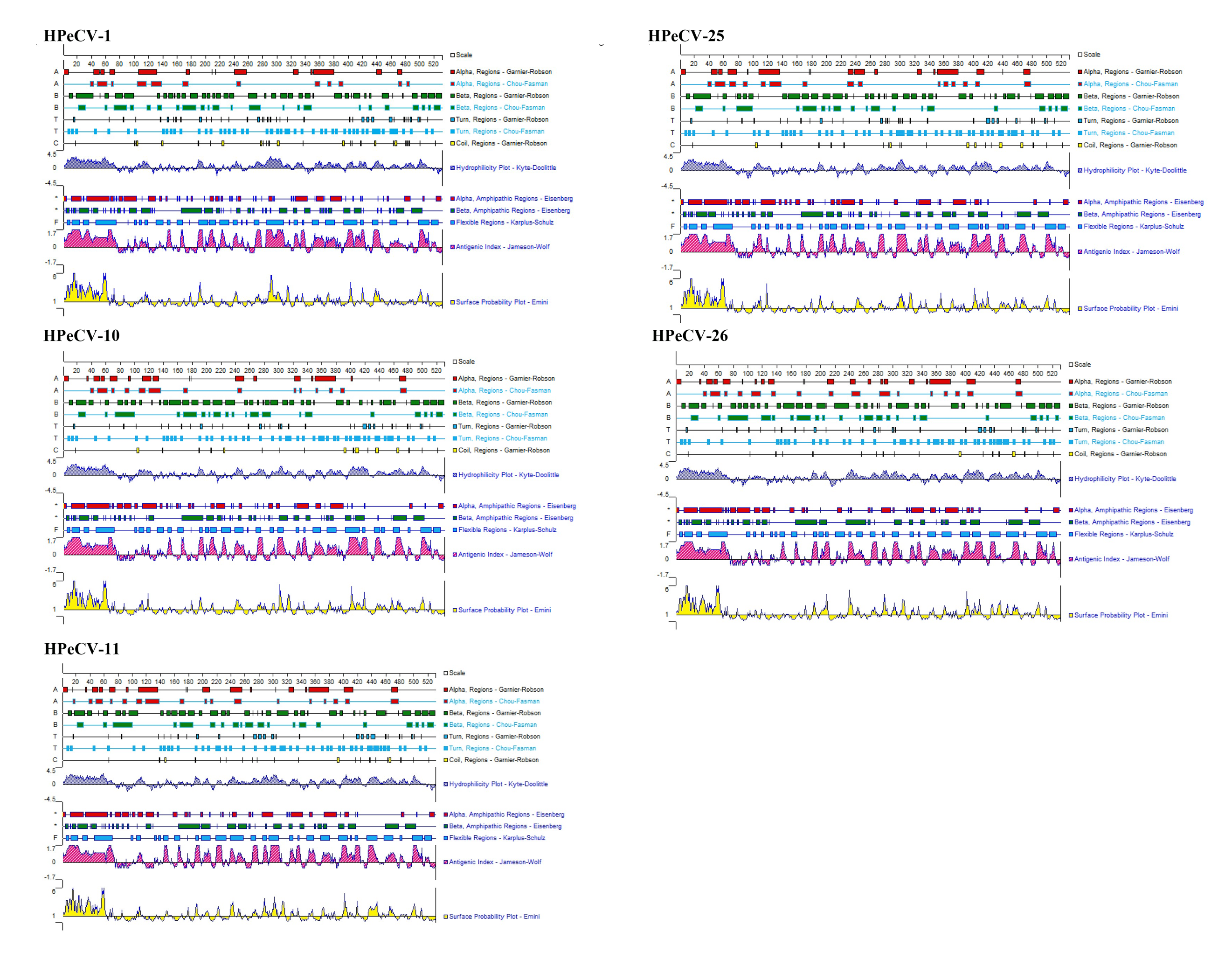


Figure S3. The structural characteristics of the novel HPeCVs predicted by PROTEAN software.

**List of accession numbers of all sequences**

|  | **Accession number** |
| --- | --- |
| HPeCV-1 | MT482428 |
| HPeCV-10 | MT482429 |
| HPeCV-11 | MT482430 |
| HPeCV-25 | MT482431 |
| HPeCV-26 | MT482432 |
| Geminiviridae | MK032758 |
| Genomoviridae | MN928911 |
| Circoviridae_Cyclovirus | HQ638060 |
| Circoviridae_Circovirus | AY035820 |
| Smacoviridae | MT138088 |
| Brisavirus_AA | MK059754 |
| Brisavirus_II | MK059755 |
| Brisavirus_MD | MK059756 |
| Vientovirus_MW | MK059772 |
| Vientovirus_XM | MK059771 |
| Vientovirus_MC | MK059770 |
| Vientovirus_LZ | MK059769 |
| Vientovirus_EC | MK059768 |
| Vientovirus_AV | MK059767 |
| Vientovirus_LT | MK059766 |
| Vientovirus_JY | MK059765 |
| Vientovirus_JB | MK059764 |
| Vientovirus_FB | MK059763 |
| Vientovirus_ES | MK059762 |
| Vientovirus_DC | MK059761 |
| Vientovirus_AL | MK059760 |
| Brisavirus_VW | MK059759 |
| Brisavirus_YH | MK059758 |
| Brisavirus_RC | MK059757 |
